# Dual roles of nanocrystalline cellulose extracted from jute (*Corchorus olitorius* L.) leaves in resisting antibiotics and protecting probiotics†

**DOI:** 10.1039/d3na00345k

**Published:** 2023-08-18

**Authors:** Yanchun Deng, Jiangpeng Pan, Xiai Yang, Sa Yang, Haiyang Chi, Xiushi Yang, Xiaoxin Qu, Shitao Sun, Linfeng You, Chunsheng Hou

**Affiliations:** a Institute of Bast Fiber Crops, Chinese Academy of Agricultural Sciences Changsha 410205 P. R. China houchunsheng@caas.cn; b Institute of Apicultural Research, Chinese Academy of Agricultural Sciences Beijing 100093 P. R. China; c Graduate School of Chinese Academy of Agricultural Sciences Beijing 100081 P. R. China; d Department of Food and Biotechnology Engineering, Chongqing Technology and Business University Chongqing 400067 P. R. China youlf@ctbu.edu.cn

## Abstract

Antibiotics can cure diseases caused by bacterial infections, but their widespread use can have some side effects, such as probiotic reduction. There is an urgent need for such agents that can not only alleviate the damage caused by antibiotics, but also maintain the balance of the gut microbiota. In this study, we first characterized the nanocrystalline cellulose (NCC) extracted from plant jute (*Corchorus olitorius* L.) leaves. Next, we evaluated the protective effect of jute NCC and cellulose on human model gut bacteria (*Lacticaseibacillus rhamnosus* and *Escherichia coli*) under antibiotic stress by measuring bacterial growth and colony forming units. We found that NCC is more effective than cellulose in adsorbing antibiotics and defending the gut bacteria *E. coli*. Interestingly, the low-dose jute NCC clearly maintained the balance of key gut bacteria like *Snodgrassella alvi* and *Lactobacillus* Firm-4 in bees treated with tetracycline and reduced the toxicity caused by antibiotics. It also showed a more significant protective effect on human gut bacteria, especially *L. rhamnosus*, than cellulose. This study first demonstrated that low-dose NCC performed satisfactorily as a specific probiotic to mitigate the adverse effects of antibiotics on gut bacteria.

## Abstract 2

Antibiotics can cure diseases caused by bacterial infections, but their widespread use can have some side effects, such as probiotic reduction.

## Graphical abstract

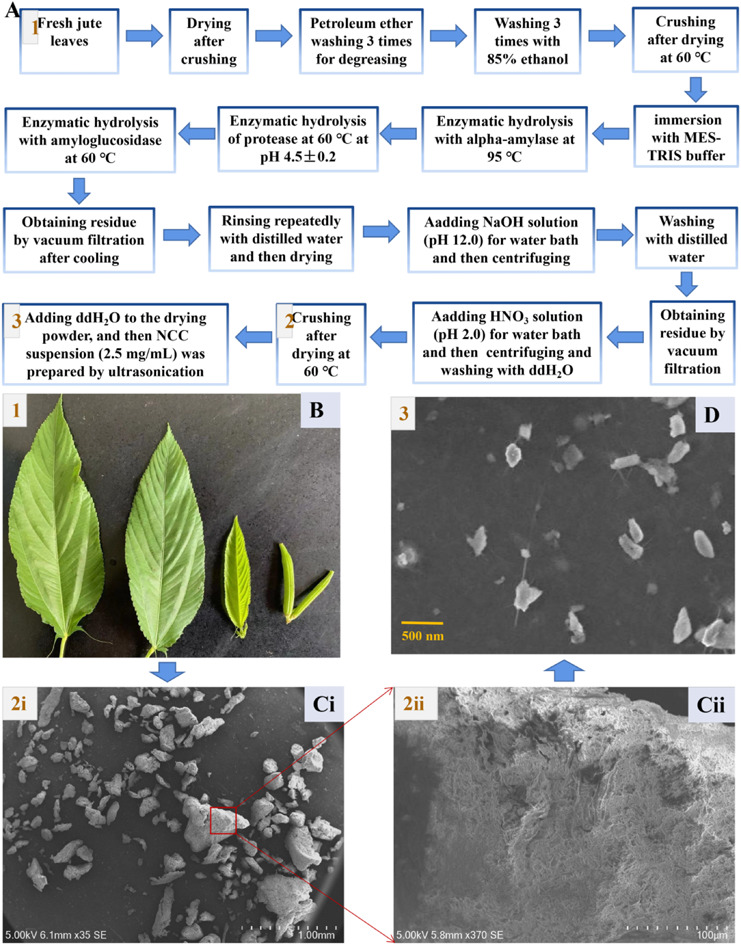

## 1. Introduction

Antibiotics have long been regarded as the most valuable drugs after the discovery of penicillin in World War II due to their high potency in treating infectious diseases.^1^ Penicillin, enrofloxacin, tetracycline, streptomycin and kanamycin are the most commonly used veterinary and human drugs.^1,2^ Antibiotics are used to fight pathogens, but they also attack commensal bacteria, disrupting the composition of intestinal microbiota and causing dysbiosis and disease.^3^ The collateral damage to intestinal microbiota caused by antibiotics has recently received increasing attention.^4^ Some studies have highlighted the interaction between antibiotic-induced long-term microbiota changes and various allergic, metabolic, immunological, and inflammatory dysfunctions.^5–7^ The gut microbial diversity is reduced after ingesting antibiotics, and full restoration of the original bacterial community is rarely achieved.^8^ Currently, drugs and dietary fiber are the most commonly used strategies to treat antibiotic damage. For example, quercetin, a representative polyphenol, has a broad spectrum of properties (including antioxidant and anti-inflammatory), and has been reported to be effective in recovering the gut microbiota in mice after antibiotic treatment and to act as a prebiotic in combatting gut dysbiosis.^9^ However, the bioavailability of polyphenols was low, and their beneficial effects and role in regulating the risk of high-prevalence diseases are difficult to demonstrate due to the high variability in bioactive effects and the complexity in the gut microbiota.^10^ Dietary fiber, a typical diet regimen with indigestible properties, can mitigate antibiotic damage.^11^ However, high doses of dietary fiber must be effective and impair the absorption of inorganic salts, fat, proteins, certain trace elements and fat-soluble vitamins.^12,13^ Thus, developing more effective probiotics or food additives with few side effects to treat antibiotic damage is becoming urgent.

Recently, some studies have been published on polysaccharides alleviating antibiotic-associated damage. *Panax quinquefolius* polysaccharides have been reported to ameliorate antibiotic-associated diarrhoea induced by antibiotics in rats.^14^ Cellulose, a natural non-starch and indigestible polysaccharide, is a glucan polymer, and one of its primary functions may be the protection of gut bacteria.^1,15^ Nanocrystalline cellulose, with a particle size of less than 600 nm, has been widely used in disease treatment and food supply in recent years. Compared to cellulose, it has higher adsorption capacity, excellent reinforcing ability, and biodegradable and biocompatible properties.^16–19^ Although Wang *et al.* found that cotton NCC can effectively treat constipation *via* altering the composition of the gut microbiota, which requires a low dose and has no effect on the organs and gut of mice,^20^ there are few reports on the protection of intestinal microbiota by NCC. Jute (*C. olitorius* L.), a perennial herb in ancient times, has been popularly used as an edible vegetable and cited in folklore as having a gastric protector.^21^ Jute leaves are said to possess stimulant, demulcent, laxative, appetizing and gastric properties, and have traditionally been used to treat fever, constipation, dysentery, liver disorder and dyspepsia.^22^ In addition, jute leaf fiber, one of the major under-utilized agricultural raw materials, consists of a mass of cellulose, followed by a small amount of hemicellulose and lignin, which makes jute an ideal source of pure nanocellulose and nanomaterials for various applications.^23,24^ These results suggest the reliability and possibility of jute NCC as a new probiotic source to be investigated. Due to macromolecular characterization, nanocellulose can also be used in the packaging of food, cosmetics, and even drug delivery. With regard to nanomaterials, it is believed that early consideration of product safety and edibility is necessary in order to realize the full potential of jute NCC uses in food-related products.

In this study, we described the preparation process of NCC from raw jute leaves by employing sequential alkali and acid reactions, enzymatic catalyzation and ultrasonication. Its shape, particle size, structural composition, and characteristics of dispersion were all identified. Then, using model human gut bacteria *in vitro* and honey bees as the model animal *in vivo*, its biological protective action on gut bacteria against antibiotic damage was examined. Our results firstly demonstrated that the NCC has a greater advantage over cellulose in terms of physicochemical properties and interactions with biological systems to mitigate the adverse effects of antibiotics on gut flora. This study provides a novel application in gut health products that double as probiotic and antibiotic alternatives.

## 2. Materials and methods

### 2.1 Jute leaves, gut bacteria and honey bees

Jute leaves for nanocrystalline cellulose extraction were obtained from the National Bast Fiber Crop Germplasm Nursery of Institute of Bast Fiber Crops, Chinese Academy of Agricultural Sciences (IBFC, CAAS, Changsha, China). The human gut bacterial strains, *E. coli* DH5 (B528413), were generously donated by Shanghai Sangon Bio-Tech (Shanghai, China), and *L. rhamnosus* isolated from Biostime Probiotic Powder was purchased from BIOSTIME (Guangzhou, China). The identification of isolated *L. rhamnosus* was determined using the 16S rRNA gene by PCR analysis according to a previous study.^25^ Honey bees were provided by the Institute of Apicultural Research, Chinese Academy of Agricultural Sciences (IAR, CAAS, Beijing, China). Healthy bees were collected from three different colonies at an experimental apiary, and newly emerged bees were obtained from brood frames and kept in an incubator at 30 °C and 60% relative humidity (RH) for 12 h until tetracycline treatment.

### 2.2 Preparation of nanocrystalline cellulose from jute leaves

It has been reported that enzymatic pretreatments of cellulosic fibers prior to the refining process have positive effects on improving the water retention value (WRV), reducing energy consumption and increasing fiber binding.^26^ Specifically, enzymes can decompose starch, pectin, protein and other non-targeted components during extracting.^27^ The jute NCC was obtained through three steps including chemical refining, enzymatic hydrolysis and ultrasonic crushing modified according to a published article.^28–30^ In brief, the dietary fibers were extracted from jute leaves according to the national standard method (China, GB 5009.88-2014). Then, the enzyme-hydrolyzed fibers were soaked in NaOH solution (pH 12.0) (w/v, 1 : 10) at 100 °C for 2 h, and the alkaline-hydrolyzed solution was neutralized to pH 7 after several cycles of distilled water washing and centrifugation. The alkaline-hydrolyzed fibers were then immersed in HNO_3_ solution (pH 2.0) (w/v, 1 : 10) for 2 h at 100 °C, and then the pure fibers were collected after several cycles of distilled water washing and centrifugation. The jute cellulose was produced after the pure fibers were dried at 55 °C and cut into small pieces. The cellulose (1 g) was dispersed in sterilized and deionized water (200 mL) and crushed with an ultrasonic cell disruptor (JY92-IIN, Ningbo Scientz Biotechnology Co., Ltd, China) with a power of 360 W for 20 min at an ice bath to avoid overheating, and the jute nanocrystalline cellulose suspension was finally prepared. The jute NCC suspensions were further used for *in vitro* bacteria and *in vivo* animal experiments. For the former, a certain amount of NCC suspension was added and diluted in LB broth and MRS broth containing *E. coli* and *L. rhamnosus*, respectively. The final NCC concentrations in LB broth and MRS broth were 0.5, 5, and 50 μg mL^−1^ according to a previous study.^31^ For the latter, a quantity of the jute NCC suspension was added and diluted in 50% sucrose solution to final concentrations of 1, 10, and 100 μg mL^−1^.

### 2.3 Scanning electron microscopy (SEM)

The morphological structure of NCC was observed using a scanning electron microscope (SEM) (SU3500, Hitachi, Japan) at an acceleration voltage of 5 kV. Furthermore, the cellulose and NCC were freeze-dried, and then observed under a SEM at an acceleration voltage of 15 kV. All samples were coated with gold before SEM observation.

### 2.4 Fourier transform infrared spectroscopy (FTIR)

The Fourier Transform Infrared Spectroscopy (FTIR) spectra of jute NCC were recorded with a FTIR spectrometer (Nicolet IS10, Thermo Fisher Scientific, USA) equipped with a damped total reflectance accessory. Before testing, the samples were ground with potassium bromide (KBr) and then subjected to precipitate. The spectra of NCC were collected in a range of 4000–400 cm^−1^, held at a resolution of 4 cm^−1^ and calculated as an average of 32 scans.

### 2.5 Particle size distribution

The particle size distribution and zeta potential measurements were conducted using a BeNano 180 Zeta (Dandong Bettersize Instruments Ltd China). The NCC samples (5 mg mL^−1^) were diluted in deionized water at a proportion of 1 : 1000 (v/v). The particle size of the diluted NCC in suspension was determined using the dynamic light scattering technique (DLS). According to Morais *et al.*, it is important to note that the results could not be completely related to the length of the NCC since the DLS technique is based on measuring an equivalent sphere diameter.^32^

### 2.6 Cytotoxicity assessment of jute NCC

A BHK21 (Baby Hamster Syrian Kidney) cell was stored at IBFC and cultured in Dulbecco's modified Eagle's medium (DMEM) supplemented with 10% (v/v) fetal calf serum, and antibiotics (0.05 U penicillin mL^−1^ and 0.05 U streptomycin mL^−1^) at 37 °C with 5% CO_2_. The BHK21 cell was prepared by the standard method and was suspended in DMEM to give 1 × 10^5^ cells mL^−1^ in six-well plates or 96-well plates and incubated overnight. Then, the medium was replaced with fresh NCC-containing medium at final concentrations of 0, 0.2, 0.39, 0.78, 1.56, 3.12, 6.25, 12.5, 50, and 100 μg mL^−1^. Morphological changes were observed under a microscope (Leica DMi8, Germany) after incubation for 24 h. Cell Counting Kit-8 (CCK-8) assay (Beyotime Biotechnology, Shanghai, China) was used to evaluate cell viability in 96-well plates. BHK21 cells were pretreated with a NCC concentration of 10 μg mL^−1^ for 24 h in six-well plates for RNA extraction. The expression level of apoptosis-related genes was detected using special primers.^33^

### 2.7 Bacterial experiments *in vitro*

Probiotic *L. rhamnosus* and enteric *E. coli*, Gram-positive and Gram-negative bacteria respectively, are usually used as model strains of gut bacteria.^31,34,35^ Two stored strains were revived according to a previously published article in broth with agar.^31^ Subsequently, one single colony isolated of each strain was cultivated in liquid broth. *L. rhamnosus* was incubated in MRS broth under static conditions at 30 °C, and *E. coli* was incubated in LB broth at 200 rpm and 37 °C. The overnight cultured broths were investigated using a spectrophotometer (UV-2700, Shimadzu, China) to measure the optical density at 600 nm (OD_600_).

For *E. coli* under kanamycin treatment, 200 μL overnight-cultured LB broth with *E. coli* was added to 50 mL fresh LB broth for 24 groups. Eight experimental treatments were designed in triplicate. The 4 groups were prepared as follows: 1000 μL of fresh LB broth, NCC (final concentration: 50 μg mL^−1^), and cellulose (final concentration: 50 μg mL^−1^) and kanamycin (final concentration: 10 μg mL^−1^) were added to the one-hour-cultured *E. coli* broth (50 mL), respectively. Another two groups were: two mixtures of 2 μL of kanamycin and NCC or cellulose suspensions were pre-incubated in a shaker at 37 °C and 40 rpm min^−1^ speed for 1 h, and then each mixture was added into the one-hour-cultured *E. coli* broth (50 mL). The last two groups were prepared as follows: 2 μL of kanamycin was added to the one-hour-cultured *E. coli* broth (50 mL), incubating for 1 h, and then NCC or cellulose was added to the two-hour-cultured *E. coli* broth (50 mL). All the cultures were incubated in an incubator (ZQPL-500AT, Laibote Technology Co., Ltd, Tianjin, China) at 37 °C and 200 rpm min^−1^. Bacterial growth was monitored and measured on the OD_600_ at different time points using a spectrophotometer. Each sample was run in triplicate and growth curves were plotted according to the OD_600_ value. All cultures of 50 mL were incubated for 24 hours, and 100 μL overnight-cultured LB broth with *E. coli* was removed and diluted serially in fresh LB broth (1 : 100), and 20 μL of the diluted sample was spread onto LB agar plates at 37 °C for 18 hours. After incubation, CFUs (Colony Forming Units) were counted for all 24 plates from the eight treatments and the average of CFUs of the control and treatment groups was compared with each other to assess the effect of the jute NCC on *E. coli* numbers. Likewise, ampicillin treatments employed a similar protocol to kanamycin treatments in *E. coli*.

For *L. rhamnosus* under ampicillin treatments, ten treat groups were designed in triplicate, and 200 μL 24-hours-cultured MRS broth with *L. rhamnosus* was added into 50 mL fresh MRS broth. Then, ampicillin (50 μg mL^−1^) was added to the six-hours-cultured MRS broth (100 mL) for different groups, respectively. Simultaneously, 1000 μL of different concentrations of NCC and cellulose (final concentration: 0, 0.5, 5, and 50 μg mL^−1^) were added to the six-hours-cultured broth (100 mL), respectively, continuing to culture statically for 42 hours at 37 °C. Bacterial growth was monitored by measuring the OD_600_ every 12 hours 6 times using a spectrophotometer. After that, each 48-hour-cultured broth with *L. rhamnosus* was diluted serially in MRS broth (1 : 1000), and 20 μL of each serially diluted sample was spread onto MRS agar plates, culturing at 37 °C for 48 hours and the CFUs were counted. Likewise, kanamycin treatments were employed with a similar protocol to ampicillin treatments in *L. rhamnosus*.

### 2.8 RNA extraction from *L. rhamnosus* and quantitative real-time PCR (qPCR) analysis

20 mL of 24- and 48-hours-cultured broth with *L. rhamnosus* under ampicillin and NCC treatments were collected by centrifugation at 12 000 rpm for 2 min at 4 °C. The total RNA of samples was extracted using a RNAprep Pure Cell/Bacteria Kit (Tiangen, Beijing, China). RNA quality was measured using a NanoDrop spectrophotometer (Thermo Electron). Approximately 500 ng RNA was reverse transcribed using a PrimeScript RT Reagent Kit with a gDNA Eraser (TaKaRa, Dalian, China) based on the manufacturer's instructions. qPCR was performed using 1 μL of 5 times diluted cDNA as the template and 0.5 μL each of the forward and reverse primers (10 mM) in a 25 μL reaction system in a 200 μL tube under a CFX96 Touch Real-Time PCR Detection System (Bio-Rad, USA) with a ChamQ Universal SYBR qPCR Master Mix (Vazyme Biotech Co., Ltd, Nanjing, China) following the manufacturer's instructions. The relative expression levels of *antitoxin* and c*haperone protein hsp 60* genes were quantified by qPCR with specific primers,^36,37^ and normalized with the 16S rRNA gene using the 2^−ΔΔCT^ method.^37^

### 2.9 Animal experiments *in vivo*

Honey bees are used as an excellent model organism for studying the function of host-associated intestinal microbiota, which is severely affected by tetracycline.^38,39^ The adult worker bee (*Apis mellifera*. L.) colonies were maintained by the standard beekeeping practice under the guideline of IAR, CAAS, as previously described in the study of Deng *et al.*^40^ The brood frames from three different colonies in IAR were transferred into an incubator (30 ± 1 °C and 60% relative humidity), the newly emerged honey bees within 24 hours were collected for the subsequent experiments. These honey bees were seemingly identified as healthy and free from bacterial diseases according to Rusenova *et al.* described.^41^ Then, about 30 newly emerged honey bees were transferred into a standard wooden cage (8 cm × 6 cm × 12 cm), and kept in an artificial climate incubator (MGC-800HP, shanghai, China), feeding with 2 mL filter-sterilized 50% sucrose every day, three replicates for each treatment. After one day, 1000 μg mL^−1^ tetracycline dissolved in filter-sterilized 50% sucrose solution was used to feed the newly emerged honey bees as described in a published paper with slight modification to ensure the removal of most gut bacterial populations.^38^ The bees of the control groups were fed sterilized 50% sucrose solution. After two days, the bees were given a steady dose of tetracycline for 2 days using jute NCC solutions in three concentrations (high, 100 μg mL^−1^; medium, 10 μg mL^−1^; low, 1 μg mL^−1^) that were dispersed in 50% filter-sterilized sucrose. Then, bees continued to be feed with the filter-sterilized 50% sucrose solution with NCC for 2 days, followed by the filter-sterilized 50% sucrose solution for 1 day. Bee mortality was also recorded daily, and any dead bees were immediately removed from the cage. This study was performed in strict accordance with the guidelines for the care and use of laboratory animals (No. SCXK 2018-0013) and approved by the Animal Ethics Committee of Institute of Bast Fifer Crops, Chinese Academy of Agricultural Sciences (CAAS, Beijing, China).

### 2.10 RNA extraction from honey bees and qPCR analysis

After 5 days, 5 bees were sampled from each treatment group (one from each cup cage), placed in 100% ethanol, and immediately stored at 4 °C. Then the midgut and hindgut were dissected as described in a previously reported paper.^42^ Dissection was performed with flame-sterilized forceps under aseptic conditions. The total RNA from 5 bees treated with tetracycline and NCC was extracted using a TRIzol Kit (Ambion, Life Technologies, USA) following the manufacturer's instructions. RNA quality was measured using a NanoDrop spectrophotometer and approximately 1000 ng RNA was reverse transcribed using a PrimeScript RT Reagent Kit with a gDNA Eraser (TaKaRa, Dalian, China) based on the manufacturer's instructions. qPCR was performed according to the method mentioned above. The relative expression levels of the immune- and detoxification-related genes were quantified by qPCR with specific primers and normalized with *actin* using the 2^−ΔΔCT^ method.^43^

### 2.11 DNA extraction from honey bee gut feces and qPCR analysis

Microbial genomic DNA was extracted from the frozen gut feces of 5 honey bees treated with tetracycline and NCC using an E.Z.N.A. Stool DNA Kit (Omega Bio-Tek, USA) following the manufacturer's instructions. Thereafter, a NanoDrop spectrophotometer (Thermo Electron) was used to measure the concentration of each sample three times. A 50 μL DNA (1 μg μL^−1^) mixture was heated at 95 °C for 3 min and then was immediately put on ice. The extracted microbial genomic DNA was used to quantify the abundance of bacteria by qPCR with specific bacterial and fungal species primers.^44,45^ qPCR was carried out using a ChamQ Universal SYBR qPCR Master Mixon under a CFX96 Touch Real-Time PCR Detection System. The absolute bacterial abundance was quantified using the absolute quantification method. Following tetracycline or the combination treatment of NCC and tetracycline, the relative abundances of core gut bacteria (*S. alvi*, *L.* Firm-4, *L.* Firm-5, *Bifidobacterium asteroids*, *Bartonella apis*, *Frischella perrera* and *G. apicola*, as well as gut fungi) from the bees were normalized with 16S using the 2^−ΔΔCT^ method.

### 2.12 Statistical analysis

Data were analyzed using one-way analysis of variance (ANOVA) and Tukey's multiple comparisons using GraphPad Prism 8 (GraphPad Software, San Diego, CA, USA). The survival rates of the bees from different groups were statistically analyzed using Log Rank Test Statistics. The mean of gene expression levels was calculated using Microsoft Excel. *P*-values below 0.05 and 0.01 represent significant and extremely significant differences, respectively.

## 3. Results and discussion

### 3.1 Preparation of jute nanocrystalline cellulose

**Fig. 1:**
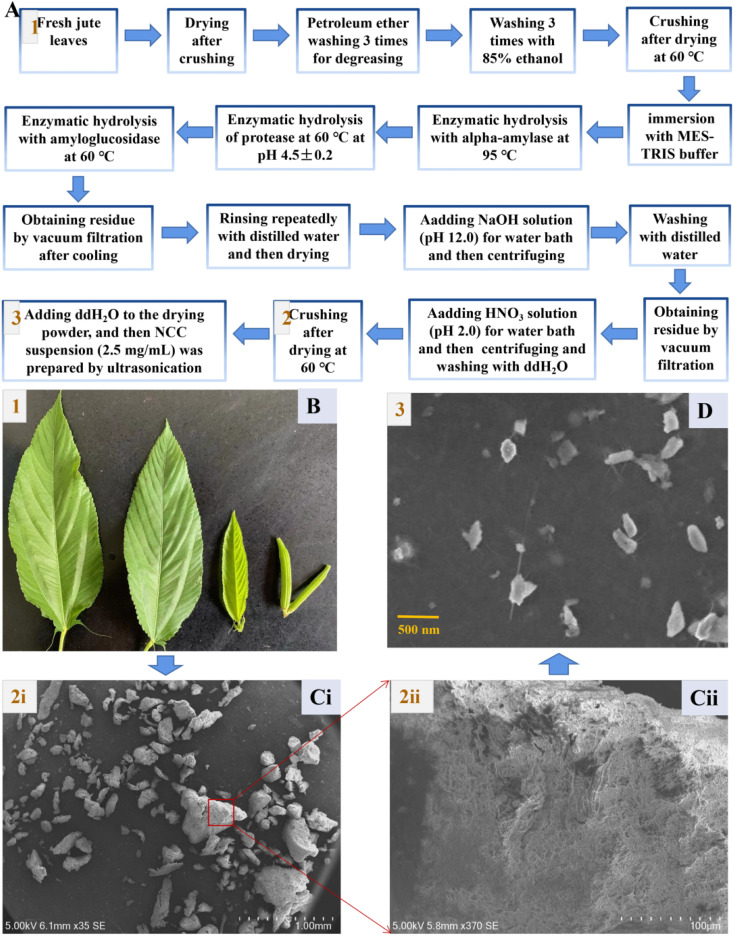
Preparation of nanocrystalline cellulose from fresh jute leaves. (A) The process of the preparation of nanocrystalline cellulose (NCC) from fresh jute leaves. (B) Images of the fresh jute leaves. (C) The SEM micrographs of cellulose. (D) The SEM micrographs of NCC.

Jute nanocrystalline cellulose (NCC) particles were obtained by processing with 15% sodium hydroxide (NaOH), followed by treatments with sodium chlorite (NaOCl_2_), H_2_O_2_ solution, 15% aqueous NaOH solution, 60% (v/v) H_2_SO_4_, ice-cold water and dialysis as described by Rabbi.^29^ By contrast, we successfully extracted edible dietary fiber from jute leaves that had been washed with petroleum ether and 85% ethanol, and then hydrolyzed with alpha-amylase, protease, and amyloglucosidase in that order (Fig. 1A and B). Raw jute fibers contain cellulose, hemicellulose, and lignin components.^23^ A food ingredient must be resistant to stomach acidity, unable to undergo enzymatic hydrolysis, and incapable of gastrointestinal absorption in order to be considered a potential prebiotics.^33^ Thus, the followed chemical processing could remove most hemicellulose and lignin, and a coupled acid treatment (HNO_3_) helped to improve the purity of the jute cellulose further. Cellulose with diameters of about 5–500 μm was finally obtained as shown in Fig. 1C(i), and had rough tuft-like surfaces (Fig. 1C(ii)). The obtained cellulose was crushed ultrasonically to produce nanocrystalline cellulose (Fig. 1D).

### 3.2 Characterization and cytotoxicity assessment of jute NCC

**Fig. 2:**
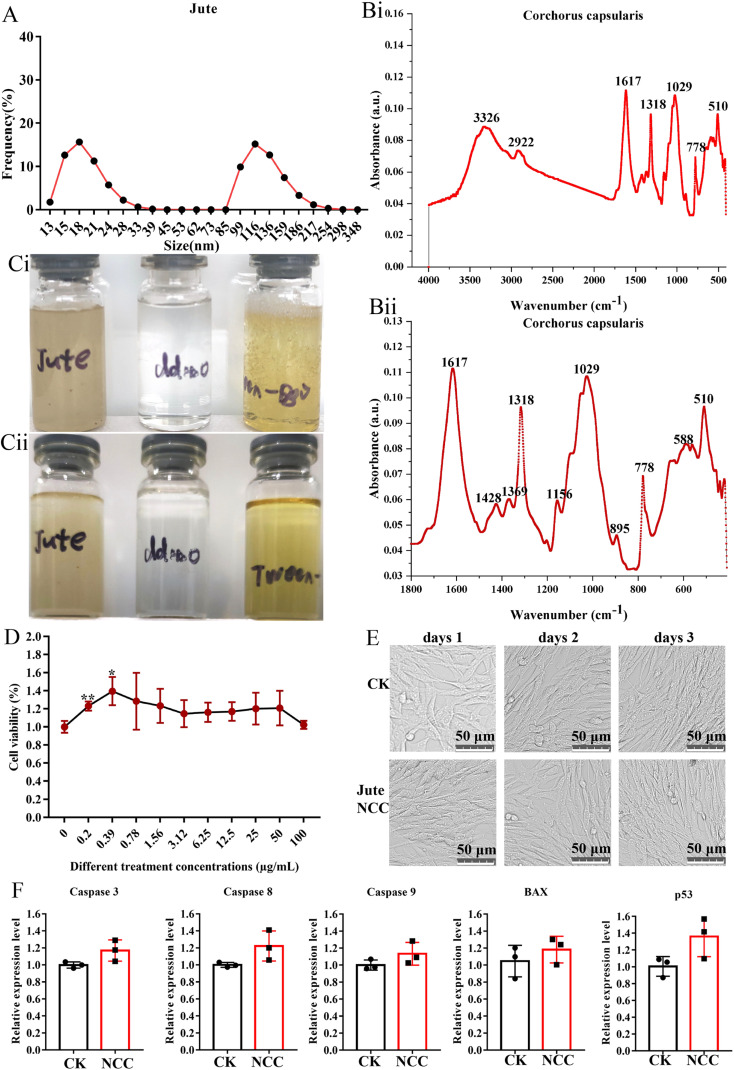
The characterization of nanocrystalline cellulose. (A) Particle size distribution of the NCC. (B) FTIR spectra of the NCC (B(i)) microparticles and the details of the region between 2000 cm^−1^ (B(i)) and 500 cm^−1^ (B(ii)). (C) Images of the NCC suspensions on days 1 (Ci) and 15 (C(ii)). (D) The viability effect of different concentrations of NCC on the BHK cell. (E) The image of the BHK cell after 10 μg mL^−1^ NCC treatment. (F) The relative expression level of the apoptosis-related gene in the BHK cell after 10 μg mL^−1^ NCC treatment.

The mean diameter of smaller NCC particles was about 18 nm with a distribution range from 13.0 nm to 39.0 nm, and the mean diameter of larger NCC particles was about 116 nm with a distribution range from 85 nm to 348 nm (Fig. 2A). The FTIR spectra of the jute NCC are shown in Fig. 2B. The spectral bands were identified at 3326 cm^−1^ (OH stretching of intramolecular hydrogen bonds for cellulose I), 2922 cm^−1^ (CH stretch), 1617 cm^−1^ (OH bend due to adsorbed water), 1428 cm^−1^ (due to CH_2_ scissoring motion in cellulose), 1369 cm^−1^ (CH bend), 1318 cm^−1^ (CH_2_ wagging), 1156 cm^−1^ (CC ring stretching band), 1029 cm^−1^ (COC pyranose ring stretching vibration), and 895 cm^−1^ (associated with the cellulosic–glycosidic bonds) (Fig. 2B(i) and B(ii)), which was analyzed according to that described by Ditzel *et al.*^46^

Because of the residual lignin, as seen in Fig. 2C, the jute NCC suspensions were light-yellow in color.^47^ NCC was fully dispersed in sterile water at a concentration of 2.5 mg mL^−1^ (Fig. 2C(i)). After 15 days of storage, NCC suspension had similar stability to Tween-80 (positive control) and water (negative control). The stability of the NCC suspension was quite impressive (Fig. 2C(ii)). These properties lay a foundation for prebiotics to be potentially added to foods.^48^

If NCC was recognized as a valuable nanomaterial for food-related applications, there is a need to reassure the safe use of NCC in food-related products. A report demonstrated that cotton NCC could not affect the blood glucose, blood lipid, liver function, kidney function, heart, liver, spleen, lung small intestine, cecum, and rectum in normal mice.^20^ Other toxicological studies also focused on unmodified 100 μg mL^−1^ nano-sized cellulose and found no signs of toxicity on Caco-2 cells.^31^ In the present study, we examined the biological effect of jute NCC on a BHK21 cell *in vitro*. As determined by Cell Counting Kit assay, the presence of jute NCC (concentration range 0.2–100 μg mL^−1^) did not significantly affect cell viability after 24 h of exposure, and even low concentrations of NCC (concentration range 0.2–0.39 μg mL^−1^) slightly promoted cell proliferation (Fig. 2D). After 24 h of exposure to 10 μg mL^−1^ NCC, the BHK21 cells appeared normal on the microscope images (Fig. 2E). The expression levels of genes associated with apoptosis were unaffected by the presence of 10 μg mL^−1^ jute NCC (Fig. 2F). Thus, when cells were exposed to the jute NCC, no evidence of toxicity in the form of cell membrane damage was observed, suggesting that the safety and excellent biocompatibility of nano-sized cellulose, including jute NCC, are reliable.

### 3.3 Protective function of NCC on human gut bacteria *in vitro*

**Fig. 3:**
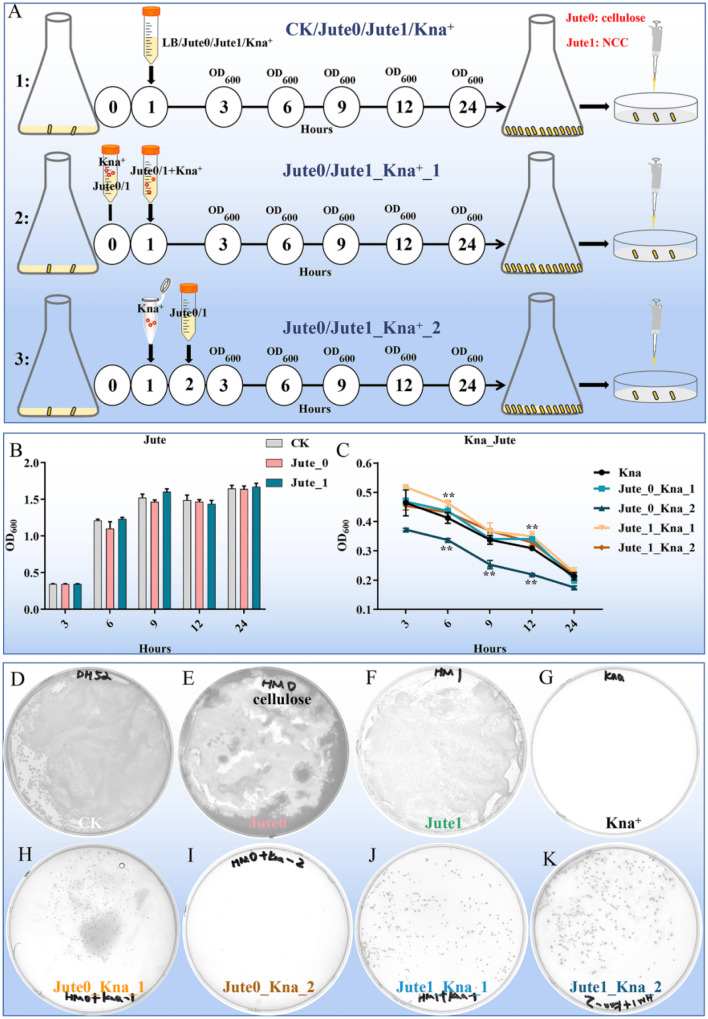
The biological potential protective effects of jute NCC on human gut bacteria (*Escherichia coli*) after kanamycin treatment. (A) Scheme and experimental procedure of the treatment of jute cellulose and NCC. (B) Effects of cellulose and NCC on growth curves of gut bacteria. (C) Effects of cellulose and NCC (50 μg mL^−1^) on growth curves of gut bacteria after kanamycin treatment (10 μg mL^−1^). (D–K) Effects of cellulose and NCC (50 μg mL^−1^) on bacterial colonies of gut bacteria after kanamycin treatment were presented on the plates for 18 h. Jute 0 represents jute cellulose; Jute 1 represents jute nanocrystalline cellulose; Kna represents kanamycin treatment. Asterisks indicate significant differences in respective genes from the control group. *P < 0.05 and **P < 0.01.

The negative effects of antibiotics are taken into serious consideration because of their inducement of long-term microbiota change, allergic symptoms, metabolic, immunological and inflammatory dysfunctions.^4^ As a vegetable, jute leaves are traditionally used to treat constipation, dysentery and dyspepsia.^22^ Therefore, the potential biological protective effects of jute NCC on human gut bacteria were investigated. As previously reported, a possible mechanism of polysaccharides to protect bifidobacteria from antibiotic inhibition is to form a viscous layer that acts as a barrier against antibiotic molecules.^1^ To assess and compare the protective effects of NCC and cellulose on human gut symbiotic bacteria (gram-negative bacteria, *E. coli*) after treatment with kanamycin, we designed three experimental groups: a pre-treatment group in which the preincubation of kanamycin and NCC or cellulose suspension was completed, and then the mixture was used to treat *E. coli*; a treatment group in which kanamycin and NCC or cellulose were simultaneously used to treat *E. coli*; a post-treatment group in which kanamycin was used to treat *E. coli*, and NCC or cellulose was added into the kanamycin-treated *E. coli* after one hour (Fig. 3A). Jute cellulose (50 μg mL^−1^) had minimal impact on the growth of *E. coli* whereas NCC had a slight boosting effect on the development of *E. coli* based on the OD_600_ values (Fig. 3B), which were plotted for bacterial growth curves at various growth stages. The pre-added cellulose and the post-added NCC had no obvious influence on the growth of *E. coli* when compared to the kanamycin treatment alone, whereas the post-added cellulose appeared to lower the bacterial concentration (Fig. 3C). Especially, pre-added NCC could dramatically increase the bacterial abundance (Fig. 3C), suggesting that jute NCC might absorb kanamycin, which was consistent with the results of Mao.^1^

The CFUs of the 8 experimental groups were counted to further analyze the differences between the groups. There was no difference in the number of *E. coli* between the control, cellulose and NCC treatment groups (50 μg mL^−1^) (Fig. 3D–F). As expected, no colonies were found on the agar plates of the kanamycin group, whereas colonies were observed in the jute cellulose and NCC groups in combination with kanamycin (Fig. 3G–K). Interestingly, compared to the cellulose post-treatment in combination with the kanamycin group (≤5 CFUs), cellulose pre-treatment showed a slight protective effect on *E. coli* after kanamycin treatment (≤200 CFUs) (Fig. 3H and I). Notably, treating it with antibiotics first and then adding NCC has a fair protective effect, but using both antibiotics and NCC at the same time will have a much better result. This indicated that, in addition to NCC absorption, NCC may also be utilized by cells (Fig. 3J and K). Additionally, we discovered that *E. coli* was protected by jute NCC (50 μg mL^−1^) following treatment with ampicillin (10 μg mL^−1^) (Fig. S1†). In addition, there was no discernible difference between the NCC-exposed and the control groups in the growth of the harmful bacteria *Staphylococcus aureus*. According to Wang *et al.*, cotton NCC could regulate gut microbiota through diversity and genus-level differences.^20^ Therefore, in contrast to the cellulose with large particles, our results once again confirmed that the NCC superior protective effect on gut symbiotic bacteria following antibiotic treatment was caused by its high dispersion in solution and adsorption on antibiotics, which was consistent with the previously reported results showing that cellulose on a nanometer scale had a better probiotic effect than cellulose of micro-size.^48^

**Fig. 4:**
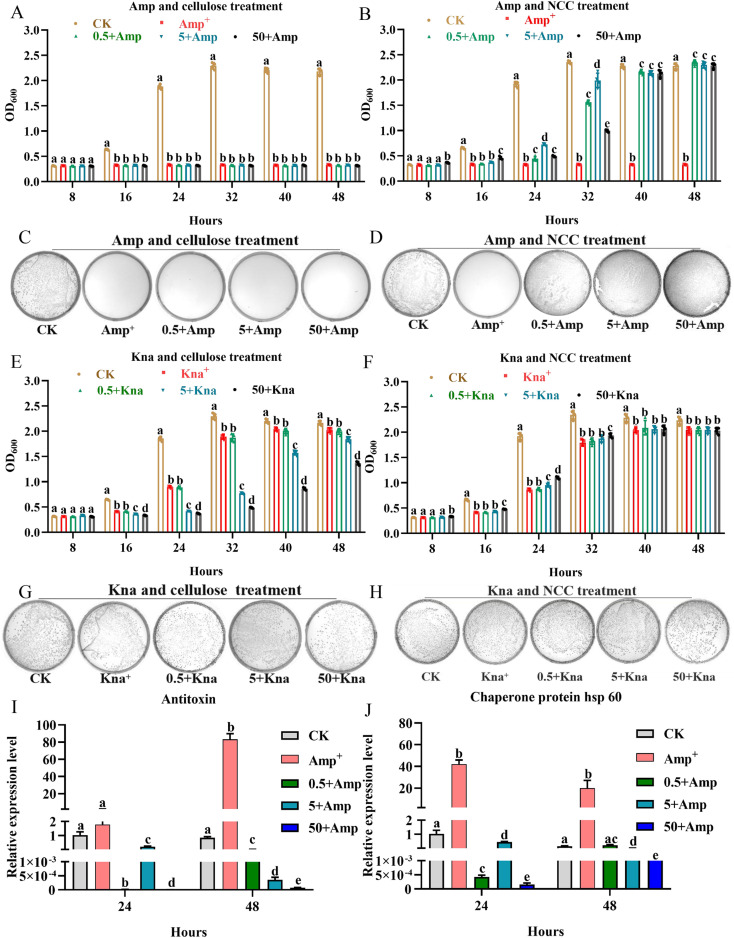
The biological potential protective effects of jute NCC on gut probiotic (*L. rhamnosus*). (A) Effects of different concentration of cellulose (0.5, 5, 50 μg mL^−1^) on the growth curves of the gut probiotic after ampicillin treatment. (B) Effects of different concentration of NCC (0.5, 5, 50 μg mL^−1^) on the growth curves of the gut probiotic after ampicillin treatment. (C) Effects of different concentration of cellulose (0.5, 5, 50 μg mL^−1^) on the CFUs of the gut probiotic after ampicillin treatment. (D) Effects of different concentration of NCC (0.5, 5, 50 μg mL^−1^) on the CFUs of the gut probiotic after ampicillin treatment. (E) Effects of different concentration of cellulose (0.5, 5, 50 μg mL^−1^) on the growth curves of the gut probiotic after kanamycin treatment. (F) Effects of different concentration of NCC (0.5, 5, 50 μg mL^−1^) on the growth curves of the gut probiotic after kanamycin treatment. (G) Effects of different concentration of cellulose (0.5, 5, 50 μg mL^−1^) on the CFUs of the gut probiotic after kanamycin treatment. (H) Effects of different concentration of NCC (0.5, 5, 50 μg mL^−1^) on the CFUs of the gut probiotic after kanamycin treatment. (I) Effects of different concentration of NCC (0.5, 5, 50 μg mL^−1^) on the expression level of antitoxin in the gut probiotic after ampicillin treatment. (J) Effects of different concentration of NCC (0.5, 5, 50 μg mL^−1^) on the expression level of HSP in the gut probiotic after ampicillin treatment. JNCC represents jute nanocrystalline cellulose, asterisks indicate significant differences in respective genes from the control group. *P < 0.05, **P < 0.01.

The gut microbiota had the ability to ferment probiotics.^49^ One typical property of a prebiotic substrate is its capacity to stimulate the growth of beneficial bacteria at low dose.^49^ Beneficial bacteria (*L. rhamnosus*) can be used to treat allergic reactions, gut-associated complications, cardiovascular health, and detoxification in humans, as well as for bee health.^50^ Therefore, the experiment was divided into three groups using low concentration cellulose or NCC (0.5, 5, and 50 μg mL^−1^), in order to examine the effects of different concentrations of cellulose or NCC on the growth curves and CFUs of *L. rhamnosus* following kanamycin or ampicillin treatment. Compared to cellulose, ampicillin treatment (50 μg mL^−1^) can directly kill *L. rhamnosus*, as shown in Fig. 4A–D, and the growth and CFUs of *L. rhamnosus* appeared to be significantly recovered by the different concentrations of jute NCC under ampicillin treatment (*P* < 0.05). Interestingly, at the time points of 24 and 32 h following ampicillin treatment, the two low concentrations of jute NCC (0.5 and 5 μg mL^−1^) had better protective potential for *L. rhamnosus* (*P* < 0.01) (Fig. 4B), indicating that NCC with low dose has a better protective effect under extremely bad conditions. There was another report that some plant functional components taken regularly might protect bacteroides species in human gut microbiota from erythromycin more effectively.^4^ However, kanamycin treatment inhibited the growth of *L. rhamnosus* but did not kill the cell. A high concentration of jute cellulose (5 and 50 μg mL^−1^) significantly inhibited the growth of *L. rhamnosus* under kanamycin treatment, while a high NCC concentration of 50 μg mL^−1^ had a slight potential protection effect on *L. rhamnosus* under kanamycin treatment at the time points of 8, 16, 24, and 32 h (*P* < 0.05) (Fig. 4E–H). These results showed that jute NCC rather than cellulose can effectively protect the gut probiotic *L. rhamnosus* under antibiotics stress, implying that jute NCC may have prebiotic potential to protect gut bacteria from antibiotic damage. Since interactions between prebiotics and gut microbes are often species-specific, we concluded that jute NCC as a potential probiotic with a wide range of sources could selectively antagonize antibiotics in the future, benefiting gut microbes but not pathogens.

In order to further evaluate the effect of NCC on the physiological process of *L. rhamnosus* under ampicillin stress, an analysis of the expression level of genes associated with function involved in antibiotic tolerance and stress response was performed. We found that the expression level of antitoxin and heat shock protein (HSP) genes was obviously up-regulated by more than 60 and 40 times in *L. rhamnosus* under ampicillin stress, respectively (Fig. 4I and J), while the different concentrations of jute NCC significantly reduced the expression level of the two genes more than 100 times in *L. rhamnosus* under ampicillin stress, and especially the high concentration of NCC significantly reduced the expression level of the antitoxin more than 10 000 times at the time points of 24 and 48 h (Fig. 4I and J). Antitoxin plays a significant role in the regulation of gene activity in most bacterial and archaeal genera, and was involved in apoptosis, antibiotic tolerance, stress response, and biofilm formation.^36,51,52^ It has been reported that temperature (48 °C) and pH (pH 4.0) stresses can stimulate the expression of antitoxin in *L. rhamnosus*,^36^ and that the transcription of HSP was induced by salt stress in *L. rhamnosus*.^37^ Consistent with our results ampicillin stress also stimulated the expression of antitoxin and heat shock protein genes. Our results suggested that jute NCC reduced the cytotoxic effects of antibiotics by decreasing the gene expression of functional associations of genes.

### 3.4 Protective function of jute NCC on honey bees *in vivo*

**Fig. 5:**
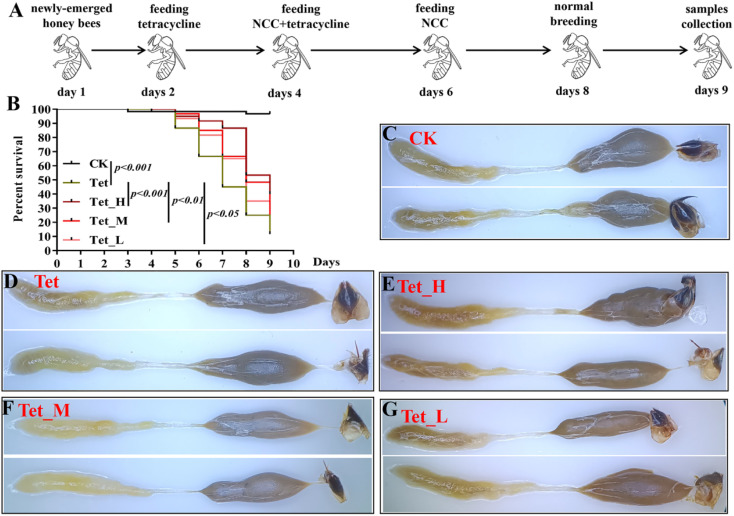
The effects of different concentrations of jute NCC on the survival rate and gut of bees after tetracycline treatment (1000 μg mL^−1^). (A) Scheme and experimental procedure of the treatment of jute NCC in tetracycline-treated bees. (B) The survival rate of bees after tetracycline or combining NCC with tetracycline treatment. (C–G) Images of the gut of bees after tetracycline or combining NCC with tetracycline treatment. (H): high, 100 μg mL^−1^; M: medium, 10 μg mL^−1^; L: low, 1 μg mL^−1^; Tet: tetracycline treatment. Asterisks indicate significant differences in respective genes from the control group. *P < 0.05 and **P < 0.01.

Our results indicated that low dose NCC can effectively treat and recover the damaged gut bacteria *in vitro*. The reported evidence that low dose cotton NCC could effectively cure constipation in mice and heal gut damage by improving gut microbiota^20^ motivated us to further investigate whether low dose jute NCC could recover gut microbiota in animals *in vivo*. Honeybees are an excellent model organism for studying host–microbiota interaction^53^ and are severely affected by tetracycline.^38,39^ Therefore, we performed *in vivo* experiments by feeding tetracycline and different dosages of jute NCC (Fig. 5A). As expected, the survival rate compared to the control group was 96.7%, and the only tetracycline treatment group had a survival rate of 11.7% on day 9, indicating that 1000 μg mL^−1^ tetracycline significantly reduced the survival rate of bees (*P* < 0.001). While the high, medium, and low concentrations of jute NCC significantly increased the survival rate after tetracycline treatment, the survival rates were 40% (*P* < 0.001), 21.7% (*P* < 0.01), and 16.7% (*P* < 0.05), respectively (Fig. 5B). Simultaneously, the high, medium, and low concentrations of jute NCC had little effect on the survival rates of the healthy bees on day 10 (Fig. S2†), indicating that there were no toxic symptoms in the healthy bees. In addition, compared to the control group, tetracycline treatment caused diarrhea in the bees (Fig. 5C and D), while the high, medium and low concentrations of NCC could effectively alleviate gut injury, especially the high concentration (Fig. 5E–G), indicating that jute NCC could restore the gut tissue damage in honey bees under tetracycline stress.

**Fig. 6:**
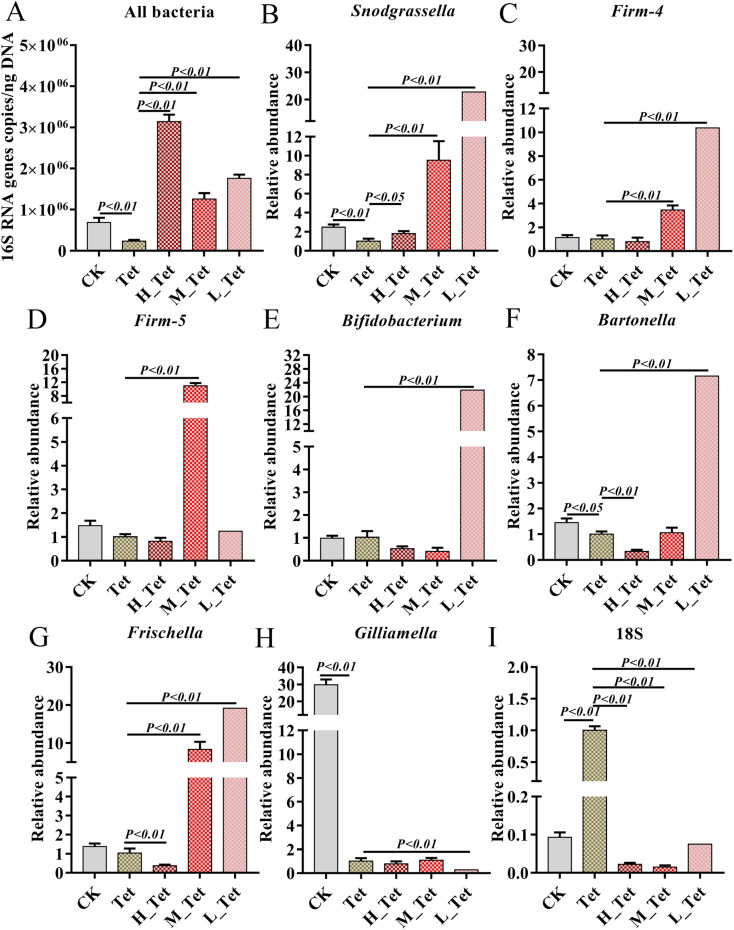
The effects of different concentrations of jute NCC on the gut microbiota of bees after tetracycline treatment (1000 μg mL^−1^). (A) The absolute abundances of gut bacterial species of bees after tetracycline or combining NCC with tetracycline treatment. (B–H) The relative abundances of core gut bacteria species (*Snodgrassella alvi*, *Lactobacillus* Firm-4, *Lactobacillus* Firm-5, *Bifidobacterium asteroids*, *Bartonella apis*, *Frischella perrera*, and *Gilliamella apicola*) of bees after tetracycline or combining NCC with tetracycline treatment. (I) The relative abundances of intestinal fungi of bees after tetracycline or combining NCC with tetracycline treatment. Asterisks indicate significant differences in respective genes from the control group. H: high, 100 μg mL^−1^; M: medium, 10 μg mL^−1^; L: low, 1 μg mL^−1^; Tet: tetracycline treatment. *P < 0.05 and **P < 0.01.

Four of eight core bacterial species in honey bee intestines were significantly affected by tetracycline with a dosage of 500 μg mL^−1^, including gram-positive *Bifidobacterium*, *L.* Firm-5 and *L.* Firm-4, and Gram-negative *S. alvi*. It has been reported that antibiotics can cause a significant change in the size of the bee gut community.^38^ Therefore, to further explore the effects of jute NCC on bee gut microbiota after tetracycline treatment (1000 μg mL^−1^), we measured the absolute abundance of gut bacteria species and the relative abundances of core gut bacteria (*S. alvi*, *L.* Firm-4, *L.* Firm-5, *Bifidobacterium asteroids*, *Bartonella apis*, *Frischella perrera*, and *G. apicola*), and the relative abundances of intestinal fungi after tetracycline or the combination of NCC with tetracycline treatment (Fig. 6). We found that the total bacterial abundance and the relative abundances of core species significantly decreased after tetracycline treatment compared to the control group. However, the relative abundances of gut fungi obviously increased in tetracycline-treated bees (Fig. 6).

Impressively, the high, medium, and low concentrations of jute NCC were able to completely and greatly enhance the absolute population of bacteria in tetracycline-treated bees, which was approximately 2–4 times higher than that of the healthy bees (Fig. 6A). These results were in line with those of Wang *et al.*,^20^ who suggested that cotton NCC might regulate the dysfunctional gut microbiota and significantly increase the relative abundance of *Lactobacillus* in mice. Interestingly, the relative abundances of *L.* Firm-5 showed a concentration-dependent-growth trait to some extent, while *S. alvi*, L. Firm-4, *B. asteroids*, *B. apis* and *F. perrera* demonstrated an opposite concentration-dependent-growth trait (Fig. 6B–G). *Lactobacillus*, a beneficial probiotic for humans, animals and honeybees,^54,55^ may promote gut motility by increasing butyric and acetic acid levels and regulate bile acids, lipid and glucose homeostasis.^56,57^*Snodgrassella* has been found to create a biofilm on the wall of the ileum, and this biofilm can act as a mechanical barrier to prevent pathogen invasion.^58^ Numerous species of *Bifidobacterium* have co-evolved with their hosts, and they demonstrate a wide spectrum of advantageous traits for their hosts.^59^ These results again suggested that jute NCC could be a valuable source of high-cellulose dietary supplement, an adjuvant to replace dietary fiber or microbial agents, and a molecule drug for the treatment of gastrointestinal diseases. However, the NCC had no protective effect on the core gut bacterium *G. apicola* but can inhibit the growth of fungi (Fig. 6H and I), although *G. apicola* may break down other potentially toxic carbohydrates and play a key role in improving dietary tolerance and maintaining the health of their bee hosts.^53,60^ Our study showed that probiotics can selectively stimulate the growth and/or activity of gut bacteria, thereby improving host health and well-being as described in a previous study.^49^ Our study expanded our knowledge on jute NCC with low doses having a better protective effect on gut bacteria against antibiotic damage. Further experimentation is required to address the regulation mechanism of the dose effect.

**Fig. 7:**
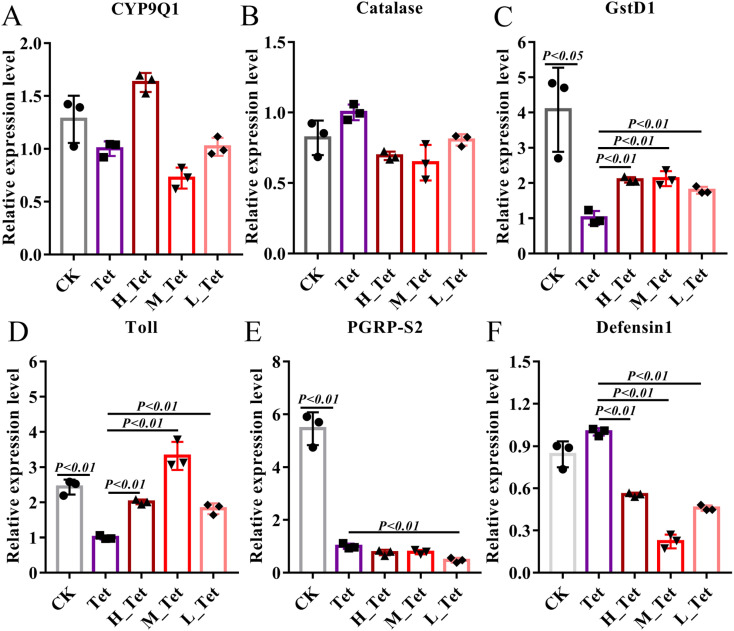
The effects of different concentrations of jute NCC on the expression level of detoxification and immune genes of bees after tetracycline treatment (1000 μg mL^−1^). Differential mRNA levels of detoxification genes, CYP9Q1 (A), Catalase (B) and Glutathione S-transferase D1 (C); immune-related genes, Toll (D), PGRP-S2 (E) and Defensin 1 (F). Asterisks indicate significant differences in respective genes from the control group. H: high, 100 μg mL^−1^; M: medium, 10 μg mL^−1^; L: low, 1 μg mL^−1^; Tet: tetracycline treatment. *P < 0.05 and **P < 0.01.

The ability of the gut microbiota to affect metabolism, regulate the gut epithelium and maintain the immune system is particularly significant.^61,62^ The adverse effects of these antibiotics are known to harm developing, cardiovascular, and metabolic systems, as well as changing immunological and anti-oxidant responses.^63^ Due to their role in antibiotic tolerance and stress response in bees, we thus used qPCR to test the expression level of detoxification genes (CYP9Q1, Catalase, and Glutathione S-transferase D1) and immune genes (Toll, PGRP-S2, and Defensin 1) after tetracycline treatment in bees (Fig. 7).^40,64^ We found that tetracycline and NCC treatment had no impact on the level of CYP9Q1 and Catalase (Fig. 7A and B), but tetracycline treatment could obviously decrease the expression level of detoxification gene Glutathione S-transferase D1 (GST) to more than 4 times (*P* < 0.05). GSTs are phase II detoxifying enzymes involved in regulating cellular antioxidants and detoxicants, maintenance of cell integrity and protection against DNA damage in a wide range of microbes, plants and animals including insects.^65^ In contrast, the high, medium, and low concentrations of jute NCC significantly increased the expression level of GSTs to 2 times after tetracycline treatment (*P* < 0.01) (Fig. 7C). Similarly, we found that tetracycline treatment could obviously decrease the expression level of the immune gene Toll and PGRPS2 (*P* < 0.01), and the three different concentrations of NCC treatment could effectively increase the expression of Toll (*P* < 0.01), but did not increase that of PGRPS2 in tetracycline-treated bees (Fig. 7D–E), suggesting that NCC treatment could effectively decrease the toxic effect on the specific immune genes in the tetracycline-treated honey bees. Our findings first confirmed that NCC has the prebiotic capacity to promote probiotic growth by showing that the expression level of the Toll-like receptor involved in the Toll signaling pathway was significantly greater after the probiotic treatment in bees.^66^ Moreover, NCC treatment significantly decreased defensin 1 expression, which can reduce the abundance of symbiotic bacteria in *Drosophila melanogaster*.^67^ Consistent with this, our study showed that NCC can significantly recover and maintain the balance of gut microbiota in bees after tetracycline treatment and reduce the expression level of defensin 1. It is therefore highlighted that further study of the effect of jute NCC on the immune system should be made.

## 4. Conclusions

The present study has revealed the better protective effects of jute NCC than cellulose on gut microbiota against some common antibiotics including kanamycin and tetracycline. One reason for the protective effect of NCC on the gut microbiota can be attributed to the adsorption of antibiotics and the formation of a viscous layer surrounding the bacteria by polysaccharides. On the other hand, jute NCC can be used as a prebiotic to replace dietary fiber for the therapy of antibiotic-related dysbiosis with good biosafety for the host. Our study firstly showed that low-dosage NCC performed better as a specific prebiotic or as an alternative for medications to mitigate the adverse effects of antibiotics on intestinal bacteria and gastrointestinal diseases, which expands the applications of nanosized plant fibers in the field of food and medical treatments. Compared to jute cellulose, NCC may both increase probiotic abundance and resistance to antibiotics, suggesting that it has a promising future prospect in various areas including food additives, drug delivery and coating for medical equipment. However, the present study demonstrated the protective effect of NCC on the human gut bacteria *E. coli* and *L. rhamnosus*, and the gut microbiota of bees. Especially, the small dosage had better prebiotic activity to protect gut beneficial bacteria against antibiotic damage. Overall, more research is needed to understand how the gut microbiota and jute NCC prebiotic interact. Future studies on the optimal dose range of NCC probiotics for each unique gut microbiome as well as the precise mechanism of dosage regularity are anticipated.

## Data availability

Data will be made available on request.

## Author contributions

Chunsheng Hou: conceptualization, supervision, project administration, and writing – reviewing and editing. Yanchun Deng: visualization, writing, methodology and software. Haiyang Chi: software and validation. Sa Yang: investigation and methodology. Xiushi Yang, Xiaoxin Qu, Shitao Sun and Jiangpeng Pan: methodology and software. Linfeng You and Xiai Yang: writing – reviewing and editing.

## Conflicts of interest

The authors declare that they have no known competing financial interests or personal relationships that could have appeared to influence the work reported in this paper.

## Supplementary Material

NA-005-D3NA00345K-s001
