# Supplementary material for: Dual roles of nanocrystalline cellulose extracted from jute (Corchorus olitorius L.) leaves in resisting antibiotics and protecting probiotics
Source: Nanoscale Adv. 2023 Aug 18;5(23):6435–48. doi: 10.1039/d3na00345k (PMC10662138; doi:10.1039/d3na00345k)

Electronic Supplementary Material (ESI) for Nanoscale Advances.
This journal is © The Royal Society of Chemistry 2023

Supplementary Information for

Dual roles of nanocrystalline cellulose extracted from jute (*Corchorus olitorius* L.) leaves in resisting antibiotics and protecting probiotics

Yanchun Deng[a†], Jiangpeng Pan[a†], Xiai Yang [a†], Sa Yang[b,c], Haiyang Chi[a], Xiushi Yang[a], Xiaoxin Qu[a], Shitao Sun[a], Linfeng You[d*], Chunsheng Hou [a*]

[a] *Institute of Bast Fifer Crops, Chinese Academy of Agricultural Sciences, Changsha 410205, P. R. China*
[b] *Institute of Apicultural Research, Chinese Academy of Agricultural Sciences, Beijing 100093, P. R. China*
[c] *Graduate School of Chinese Academy of Agricultural Sciences, Beijing 100081, P. R. China*
[d] *Department of Food and Biotechnology Engineering, Chongqing Technology and Business University, Chongqing 400067, P.R. China*

† These authors contributed equally.
* Correspondence: youlf@ctbu.edu.cn (L. You) or houchunsheng@caas.cn (C. Hou)

Supplementary figures

Fig. S1. The biological potential protective effects of jute NCC on human gut bacteria (*Escherichia coli*) after kanamycin treatment. (A) Effects of cellulose and NCC (50 μg/mL) on growth curves of gut bacteria after ampicillin treatment (10 μg/mL). (B-C) Effects of cellulose and NCC (50 μg/mL) on the bacterial colonies of gut bacteria after ampicillin treatment were presented on the plates for 18 h. Jute 0 represents jute cellulose; Jute 1 represents jute nanocrystalline cellulose; Amp represents ampicillin treatment.

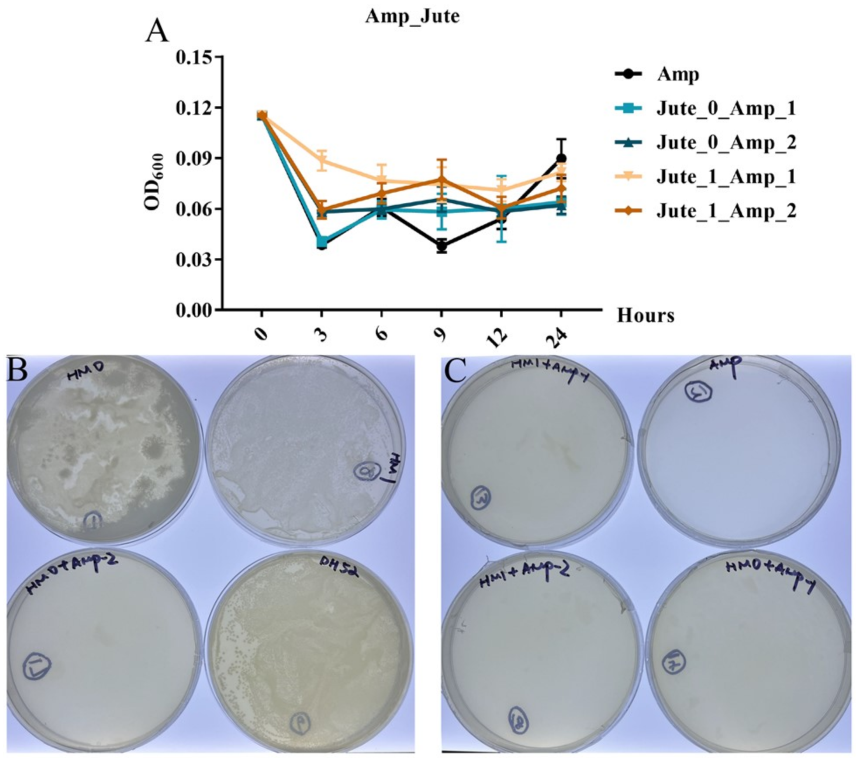

Fig. S2. The effect of different concentration of jute NCC on survival rate of honeybees. H: high, 100 μg/mL; M: medium, 10 μg/mL, and L: low, 1 μg/mL.

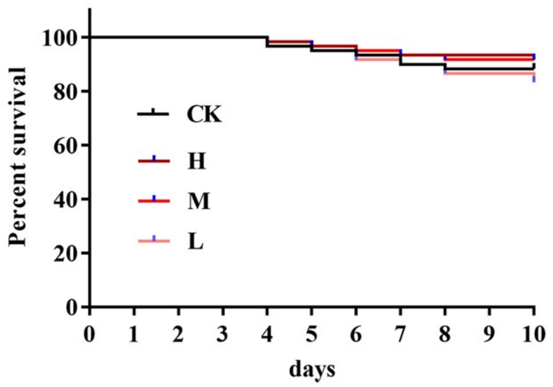

Supplement: NA-005-D3NA00345K-s001 [file NA-005-D3NA00345K-s001.pdf]
